# Supplementary material for: Differentiating Ductal Adenocarcinoma of the Pancreas from Benign Conditions Using Routine Health Records: A Prospective Case-Control Study
Source: Cancers (Basel). 2022 Dec 31;15(1):280. doi: 10.3390/cancers15010280 (PMC9818842; doi:10.3390/cancers15010280)
Supplement: Supplementary file 1 [file cancers-15-00280-s001.zip › cancers-2078416-supplementary.pdf]

Supplementary material

Contents

Table S1. A list of diagnoses that fall under the three diagnostic groups with their associated ICD-10 and ICD-0-3 codes.

Table S2. Clinical variables considered for the observational association study. Statistically significant variables were then used in the development of the risk prediction models.

Table S3. Reference ranges of the blood tests used to determine the high and low ranges for the corresponding tests.

Table S4. Frequency of diagnoses in the study patient groups

Table S5. Baseline characteristics of the study groups.

Table S6. Observed pre-diagnostic symptoms and common blood test results of the study groups.

Table S7. Modified odds of PDAC associated with different predictor variables among different participant subgroups.

Table S8. Performance measures of the final model in differentiating PDAC from various subgroups of non-malignant pancreatic conditions.

Figure S1. Forest plot showing crude association between study variables (demographic, lifestyle, comorbidity ands symptoms) and odds of PDAC in comparison to non-malignant pancreatic disease group.

Figure S2. Forest plot showing crude association between blood test result variables (a: categorical with known normal range as reference; b: categorical with interquartile of known normal range as reference; c: continuous) and odds of PDAC in comparison to non-malignant pancreatic disease blood test, interquartile of their normal range has been used as reference.

Supplementary Table S1. A list of diagnoses that fall under the three diagnostic groups with their associated ICD-10 and ICD-0-3 codes.

ICD-10	ICD-0-3	Diagnosis
**Pancreatic ductal adenocarcinoma (PDAC)**		
C25	8500/3	Infiltrating duct carcinoma NOS
**Pancreas cancers (PC)**		
C25	8550/3	Acinar cell carcinoma
C25	8551/3	Acinar cell cystadenocarcinoma
C25	8453/3	Intraductal papillary-mucinous carcinoma, invasive
C25	8470/3	Mucinous cystadenocarcinoma NOS
C25	8971/3	Pancreatoblastoma
C25	8441/3	Serous cystadenocarcinoma, NOS
C25	8452/3	Solid pseudopapillary carcinoma
C25	8240/3	Carcinoid tumor, NOS
C25	8240/3	Typical carcinoid
C25	8240/3	Bronchial adenoma, carcinoid
C25	8249/3	Atypical carcinoid tumor
C25	8246/3	Neuroendocrine carcinoma, NOS
C25	8241/3	Enterochromaffin cell carcinoid
C25	8153/3	Gastrinoma. malignant
C25	8152/3	Glucagonoma. malignant
C25	8151/3	Insulinoma. malignant
C25	8156/3	Somatostatinoma malignant
C25	8155/3	Vipoma malignant
C25	9260/3	Ewing sarcoma
C25	8806/3	Desmoplastic small round cell tumor
C25	8453/2	Intraductal papillary-mucinous carcinoma, non-invasive
C25	8470/2	Mucinous cystadenocarcinoma, non-invasive
**Non-malignant pancreatic conditions**		**(PnC)**
K85.2		Alcohol-induced acute pancreatitis
K85.3		Drug-induced acute pancreatitis
K85.0		Idiopathic acute pancreatitis
K85.9		Acute pancreatitis, unspecified
K85.1		Biliary acute pancreatitis
K86.2		Cyst of pancreas
K86.3		Pseudocyst of pancreas
K83.4		Spasm of sphincter of Oddi
Q45.3		Other congenital malformations of pancreas and pancreatic duct
C25	8150/0	Pancreatic endocrine tumor, benign
C25	8150/0	Islet cell adenomatosis
C25	8453/0	Intraductal papillary-mucinous adenoma
C25	8470/0	Mucinous cystadenoma, NOS
	8441/0	Serous cystadenoma, NOS
	8441/0	Serous microcystic adenoma
	9170/0	Lymphangioma, NOS
	8850/0	Lipoma, NOS

Supplementary Table S2. Clinical variables considered for the observational association study. Statistically significant variables were then used in the development of the risk prediction models.

Demographics	Symptoms	Medical history	Lifestyle history	Laboratory results
Gender	Pain	Diabetes	Smoking	Urea (UREA)
Age	Jaundice	Cholesterol	Alcohol	Potassium (K)
Ethnicity	Weight loss	Hypertension	Body mass index (BMI)	Sodium (NA)
	Nausea	Cardiovascular		Total calcium (CA)
	Vomiting	Chronic respiratory		Creatinine (CREAT)
	Diarrhoea	Chronic kidney		Total bilirubin (TBIL)
	Constipation	Chronic liver		Alkaline phosphatase (ALP)
	Fatigue	Cancer		Alanine aminotransferase (ALT)
	Loss of appetite			Aspartate transaminase (AST)
	Pruritus			Albumin (ALB)
	Steatorrhoea			Amylase (AMY)
	Change in bowel habits			Carbohydrate antigen (CA19-9)
				Haemoglobin (HB)
				Red blood cell count (RBC)
				Mean corpuscular volume (MCV)
				Platelets (PLT)
				White blood cell count (WBC)
				Basophils (BASO)
				Eosinophils (EOSI)
				Lymphocytes (LYMP)
				Neutrophils (NEUT)
				C-reactive protein (CRP)

Supplementary Table S3. Reference ranges of the blood tests used to determine the high and low ranges for the corresponding tests.

Blood test	Reference range	Interquartile of the reference range
Haemoglobin (HB)	130 - 180 g/L (male)<br>115 - 165 g/L (female)	142.5-167.5 g/L (male)<br>127.5-152.5 g/L (male)
White Blood Cell Count (WBC)	4.0 - 11.0 10^9 /L	5.74-9.25 10^9 /L
Red Blood Cell Count (RBC)	4.5 - 6.5 10^{12} /L (male)<br>3.8 - 5.8 10^{12} /L (female)	5-6 10^{12} /L (male)<br>4.3-5.3 10^{12} /L (female)
Mean Corpuscular Volume (MCV)	80 - 100 fL	85-95 fL
Platelet count (PLT)	150 - 450 10^9 /L	225-375 10^9 /L
Neutrophils (NEUT)	2 - 7.5 10^9 /L	3.37-6.12 10^9 /L
Lymphocytes (LYMP)	1.5 - 4.5 10^9 /L	2.25-3.75 10^9 /L
Eosinophils (EOSI)	0 - 0.4 10^9 /L	0.1-0.3 10^9 /L
Basophils (BASO)	0 - 0.1 10^9 /L	0-0.1 10^9 /L
Total bilirubin (TBIL)	1 - 17 µmol/L	5-13 µmol/L
Alkaline phosphatase (ALP)	30 - 130 U/L	55-105 U/L
Aspartate transaminase (AST)	2 - 45 U/L	12.75-34.25 U/L
Alanine transaminase (ALT)	1 - 50 U/L	13.25-37.75 U/L
Amylase (AMY)	30 - 118 U/L	52-96 U/L
Albumin (ALB)	35 - 50 g/L	38.75-46.25 g/L
CA 19-9 (C199)	0 - 37 kU/L	9.25-27.75 kU/L
C-reactive protein (CRP)	0 - 5 mg/L	1.25-3.75 mg/L
Creatinine (CREA)	50 - 120 µmol/L	67.5-102.5 µmol/L
Urea (UREA)	2.5 - 7 mmol/L	3.62-5.87 mmol/L
Sodium (NA)	133 - 146 mmol/L	136.25-142.75 mmol/L
Potassium (K)	3.5 - 5 mmol/L	3.87-4.62 mmol/L
Total calcium (CA)	2.2 - 2.6 mmol/L	2.3-2.5 mmol/L

Supplementary Table S4. Frequency of diagnoses in the study patient groups.

**Diagnosis**	**Count (%)**
**PDAC**	**344(45·1%)**
Pancreatic ductal adenocarcinoma	344
**Non-malignant pancreatic conditions (PnC)**	**360(47·2%)**
Acute	***91***
Acute pancreatitis - unspecified	32
Biliary acute pancreatitis	30
Alcohol/drug-induced acute pancreatitis	19
Idiopathic acute pancreatitis	9
Autoimmune pancreatitis	1
Chronic	***126***
Chronic pancreatitis	113
Spasm of sphincter of oddi	9
Congenital malformation of pancreas and pancreatic duct	4
Cyst/Tumour	***143***
Cyst and pseudocyst of pancreas	57
Intraductal papillary mucinous adenoma	36
Serous cystadenoma	22
Pancreatic endocrine tumor - benign	14
Mucinous cystadenoma	11
Serous microcystic adenoma	3
**Pancreas cancer**	**58(7·6%)**
Neuroendocrine Carcinoma	20
Intraductal papillary-mucinous carcinoma - non-invasive	11
Insulinoma	9
Solid pseudopapillary carcinoma	6
Intraductal papillary mucinous carcinoma	5
Mucinous cystadenocarcinoma - non-invasive	2
Glucagonoma	2
Acinar cell carcinoma	1
Carcinoid tumour	1
Serous cystadenocarcinoma	1
**Total**	**762(100%)**

Supplementary Table S5. Baseline characteristics of the study groups.

	PDAC (n=344)	PnC (n=360)	Total (N=704)	p value[a]
**Gender**				0.019
Female	162 (47.1%)	138 (38.3%)	300 (42.6%)	
Male	182 (52.9%)	222 (61.7%)	404 (57.4%)	
**Ethnicity**				< 0.001
Caucasian	272 (79.1%)	241 (66.9%)	513 (72.9%)	
South Asian	17 (4.9%)	37 (10.3%)	54 (7.7%)	
Afro-Caribbean	30 (8.7%)	27 (7.5%)	57 (8.1%)	
Other	15 (4.4%)	20 (5.6%)	35 (5.0%)	
Unknown	10 (2.9%)	35 (9.7%)	45 (6.4%)	
**Age**				< 0.001 [b]
Median (IQR)	68.0 (60.0-75.0)	55.0 (45.0-65.2)	62.0 (51.0-72.0)	
**Body mass index (BMI)**				0.002 [b]
Median (IQR)	24.2 (22.1-27.5)	25.5 (22.8-29.3)	25.0 (22.3-28.2)	
Unknown	71 (20.6%)	85 (23.2%)	156 (22.2%)	
**Smoking**				0.022
Never	160 (46.5%)	136 (37.8%)	296 (42.0%)	
Past	95 (27.6%)	94 (26.1%)	189 (26.8%)	
Current	64 (18.6%)	98 (27.2%)	162 (23.0%)	
Unknown	25 (7.3%)	32 (8.9%)	57 (8.1%)	
**Drinking**				0.018
Never	129 (37.5%)	109 (30.3%)	238 (33.8%)	
Past	44 (12.8%)	76 (21.1%)	120 (17.0%)	
Current	140 (40.7%)	140 (38.9%)	280 (39.8%)	
Unknown	31 (9.0%)	35 (9.7%)	66 (9.4%)	
**Diabetes**				0.002
Yes	120 (34.9%)	87 (24.2%)	207 (29.4%)	
**Cholesterol**				0.073
Yes	113 (32.8%)	96 (26.7%)	209 (29.7%)	
**Hypertension**				<0.001
Yes	158 (45.9%)	102 (28.3%)	260 (36.9%)	
**Cardiovascular disease**				0.054
Yes	70 (20.3%)	51 (14.2%)	121 (17.2%)	
Unknown	1 (0.3%)	0 (0.0%)	1 (0.1%)	
**Chronic respiratory disease**				0.592
Yes	54 (15.7%)	57 (15.8%)	111 (15.8%)	
Unknown	1 (0.3%)	0 (0.0%)	1 (0.1%)	
**Chronic kidney disease**				0.292
Yes	19 (5.5%)	28 (7.8%)	47 (6.7%)	
Unknown	1 (0.3%)	0 (0.0%)	1 (0.1%)	
**Chronic liver disease**				0.526

Yes	11 (3.2%)	14 (3.9%)	25 (3.6%)	
Unknown	1 (0.3%)	0 (0.0%)	1 (0.1%)	
**Past history of Cancer**				0.017
Yes	42 (12.2%)	25 (6.9%)	67 (9.5%)	

[a] Differences between groups evaluated by the $\chi 2$ test, unless otherwise stated.

[b] Differences between groups evaluated by Kruskal-Wallis rank sum test.

PDAC, pancreatic ductal adenocarcinoma; PnC: non-malignant pancreatic diseases; IQR, interquartile range

Supplementary Table S6. Observed pre-diagnostic symptoms and common blood test results of the study groups

	PDAC (n=344)	PnC (n=360)	Total (N=704)	p value
**Symptoms[a]**				
**Pain**				<0.001
Yes	175 (50.9%)	262 (72.8%)	437 (62.1%)	
**Jaundiced**				<0.001
Yes	169 (49.1%)	57 (15.8%)	226 (32.1%)	
**Weight loss**				<0.001
Yes	204 (59.3%)	140 (38.9%)	344 (48.9%)	
**Nausea**				0.009
Yes	95 (27.6%)	136 (37.8%)	231 (32.8%)	
Unknown	0 (0.0%)	1 (0.3%)	1 (0.1%)	
**Vomiting**				<0.001
Yes	62 (18.0%)	124 (34.4%)	186 (26.4%)	
Unknown	0 (0.0%)	1 (0.3%)	1 (0.1%)	
**Diarrhoea**				0.021
Yes	61 (17.7%)	93 (25.8%)	154 (21.9%)	
Unknown	1 (0.3%)	0 (0.0%)	1 (0.1%)	
**Constipation**				0.712
Yes	64 (18.8%)	78 (21.2%)	142 (20.1%)	
Unknown	4 (1.2%)	4 (1.1%)	8 (1.1%)	
**Fatigue**				0.076
Yes	25 (7.3%)	15 (4.2%)	40 (5.7%)	
**Loss of appetite**				0.910
Yes	12 (3.5%)	12 (3.3%)	24 (3.4%)	
**Pruritus**				0.005
Yes	16 (4.7%)	4 (1.1%)	20 (2.8%)	
**Steatorrhea**				0.208
Yes	9 (2.6%)	4 (1.1%)	13 (1.8%)	
Unknown	0 (0.0%)	1 (0.3%)	1 (0.1%)	
**Change in bowel habits**				0.077
Yes	88 (25.6%)	72 (20.0%)	160 (22.7%)	
**Blood tests[b]**				
**Haemoglobin (HB)**				<0.001
Median (IQR)	120.5 (100.0-132.5)	130.0 (117.0-142.0)	125.0 (106.5-39.9)	
Unknown	21 (6.1%)	53 (14.7%)	74 (10.5%)	
**Red blood cell count (RBC)**				<0.001
Median (IQR)	4.1 (3.7-4.6)	4.5 (4.2-4.9)	4.3 (3.9-4.8)	
Unknown	51 (14.8%)	100 (27.8%)	151 (21.4%)	

**Mean corpuscular volume (MCV)**				<0.001
Median (IQR)	89.4 (85.5-93.9)	87.8 (84.3-91.1)	88.9 (84.8-92.6)	
Unknown	51 (14.8%)	100 (27.8%)	151 (21.4%)	
**Platelets (PLT)**				<0.001
Median (IQR)	287.0 (227.0-376.5)	261.5 (214.8-311.2)	273.0 (219.0-351.0)	
Unknown	21 (6.1%)	56 (15.6%)	77 (10.9%)	
**White blood cell count (WBC)**				0.054
Median (IQR)	8.4 (6.8-10.4)	7.9 (6.4-9.9)	8.1 (6.7-10.2)	
Unknown	21 (6.1%)	56 (15.6%)	77 (10.9%)	
**Neutrophils (NEUT)**				0.001
Median (IQR)	5.4 (4.3-7.4)	4.9 (3.6-6.6)	5.1 (3.9-7.0)	
Unknown	51 (14.8%)	100 (27.8%)	151 (21.4%)	
**Lymphocytes (LYMP)**				<0.001
Median (IQR)	1.7 (1.2-2.3)	1.9 (1.5-2.4)	1.8 (1.4-2.4)	
Unknown	51 (14.8%)	100 (27.8%)	151 (21.4%)	
**Basophils (BASO)**				0.060
Median (IQR)	0 (0.0- 0.1)	0 (0.0-0.1)	0 (0.0-0.1)	
Unknown	51 (14.8%)	100 (27.8%)	151 (21.4%)	
**Eosinophils (EOSI)**				0.467
Median (IQR)	0.1 (0.1-0.2)	0.1 (0.1-0.3)	0.1 (0.1-0.2)	
Unknown	51 (14.8%)	100 (27.8%)	151 (21.4%)	
**C-reactive protein (CRP)**				< 0.001
Median (IQR)	20.0 (7.0-55.0)	7.0 (4.0, 38.0)	14.0 (5.0-48.8)	
Unknown	80 (23.3%)	141 (39.2%)	221 (31.4%)	
**Total bilirubin (TBIL)**				<0.001
Median (IQR)	14.5 (6.0, 47.5)	8 (5.8, 12.0)	10 (6.0, 20.0)	
Unknown	21 (6.1%)	61 (16.9%)	82 (11.6%)	
**Alkaline phosphatase (ALP)**				<0.001
Median (IQR)	162.5 (93.5, 312.8)	86.0 (69.0, 117.0)	107.0 (77.8, 212.0)	
Unknown	20 (5.8%)	60 (16.7%)	80 (11.4%)	
**Aspartate aminotransferase (AST)**				0.041
Median (IQR)	53.0 (30.0-96.2)	41.0 (23.0-73.0)	47.0 (26.0-86.0)	
Unknown	168 (48.8%)	271 (75.3%)	439 (62.4%)	
**Alanine transaminase (ALT)**				<0.001
Median (IQR)	34.2 (19.0-8.0)	21.0 (15.0-41.8)	27.0 (17.0-58.0)	
Unknown	20 (5.8%)	58 (16.1%)	78 (11.1%)	
**Albumin (ALB)**				<0.001
Median (IQR)	42 (38.0-45.0)	44.0 (41.0-47.0)	43 (39.0-46.0)	
Unknown	20 (5.8%)	56 (15.6%)	76 (10.8%)	

**Amylase (AMY)**				<0.001
Median (IQR)	43.0 (25.2-76.0)	90.0 (57.5-153.2)	61.0 (32.0-110.0)	
Unknown	254 (73.8%)	292 (81.1%)	546 (77.6%)	
**CA19-9**				<0.001
Median (IQR)	294.0 (63.8-1144.0)	15.9 (9.0-35.0)	115.2 (19.3-670.5)	
Unknown	81 (23.5%)	247 (68.6%)	328 (46.6%)	
**Total calcium (CA)**				0.089
Median (IQR)	2.3 (2.2-2.4)	2.2 (2.2-2.4)	2.3 (2.2-2.4)	
Unknown	96 (27.9%)	186 (51.7%)	282 (40.1%)	
**Sodium (NA)**				<0.001
Median (IQR)	138.5 (135.9-141.0)	141.0 (139.0-142.0)	140.0 (137.0-142.0)	
Unknown	24 (7.0%)	54 (15.0%)	78 (11.1%)	
**Potassium (K)**				0.018
Median (IQR)	4.4 (4.1-4.8)	4.3 (4.1-4.6)	4.4 (4.1-4.7)	
Unknown	24 (7.0%)	52 (14.4%)	76 (10.8%)	
**Creatinine (CREA)**				0.006
Median (IQR)	69 (56.0-84.0)	74.0 (62.0-87.0)	71 (58.5-86.0)	
Unknown	24 (7.0%)	55 (15.3%)	79 (11.2%)	
**Urea (UREA)**				0.080
Median (IQR)	5 (3.9-6.3)	4.8 (3.7-6.0)	4.9 (3.7-6.1)	
Unknown	24 (7.0%)	60 (16.7%)	84 (11.9%)	

[a] Differences between groups evaluated by the $\chi 2$ test.
[b] Differences between groups evaluated by Kruskal-Wallis rank sum test.
PDAC, pancreatic ductal adenocarcinoma; PnC: non-malignant pancreatic diseases; IQR, interquartile range

Supplementary Table S7. Modified odds of PDAC associated with different predictor variables among different participant subgroups. Only statistically significant interactions.

Modifier	Predictor		p-inter
	**OR [95% CI]**	**p-val**	
Diabetes (ref=No)	*Alanine transaminase (ref=Normal)*		0·010
	High		
Yes	0·27 [0·08-0·86]	0·04	
Smoking (ref=Never)	*Chronic liver (ref=No)*		0·006
	Yes		
Past	2·73 [0·14-54·1]	0·711	
Current	1·86E-7 [0-Inf]	0·977	
Hypertension (ref=No)	*Weight loss (ref=No)*		0·005
	Yes		
Yes	**3·82 [1·63-8·93]**	**0·005**	
Cancer (ref=No)	*Vomit (ref=No)*		0·009
	Yes		
Yes	9·29E6[0-Inf]	0·98	

OR adjusted for gender, ethnicity and age group. The reported p values are corrected for multiple testing via Benjamini-Hochberg method.
Interaction between groups evaluated by the likelihood ratio test. Only statistically significant predictor-modifier interaction pairs are shown (p value for interaction<0.01).
OR, odds ratio; CI, confidence interval.

Supplementary Table S 8. Performance measures of the final model in differentiating PDAC from various subgroups of non-malignant pancreatic conditions, using the probability thresholds obtained from maximum F_1 and F_2-score on the final model.

PDAC (N=344) vs PnC	AUROC	Maximum F_1 probability (0.42)				Maximum F_2 probability (0.15)			
		Sensitivity	Specificity	PPV	NPV	Sensitivity	Specificity	PPV	NPV
Acute (N=91)	0.88	0.81	0.79	0.94	0.52	0.97	0.40	0.86	0.77
Chronic (N=126)	0.86	0.81	0.75	0.90	0.59	0.97	0.37	0.81	0.81
Cyst (N=57)	0.87	0.81	0.75	0.95	0.39	0.97	0.32	0.90	0.62
Tumour (N=86)	0.84	0.81	0.70	0.91	0.48	0.97	0.24	0.84	0.66
All (N=360)	0.86	0.81	0.75	0.75	0.80	0.97	0.34	0.58	0.92

PDAC, pancreatic ductal adenocarcinoma; PnC: non-malignant pancreatic diseases; AUROC: Area under the receiver-operating-characteristic curve; PPV: Positive predictive value/Precision; NPV: Negative predictive value

	OR	95% CI	p val
**Gender (ref=Female)**			
Male	0.7	0.49-0.99	0.086
**Ethnicity (ref=Caucasian)**			
South Asian	0.39	0.2-0.77	0.018
Afro-Carribean	0.75	0.39-1.44	0.386
Other	0.58	0.25-1.33	0.245
**Age (ref=18-55)**			
55-64	3.54	2.1-5.99	<0.001
65-74	6.72	4.01-11.25	<0.001
75+	10.35	5.83-18.38	<0.001
**Body mass index**			
per 5 unit increase	0.86	0.73-1.01	0.097
**Smoking (ref=Never)**			
Past	0.79	0.52-1.21	0.276
Current	0.52	0.33-0.8	0.01
**Alcohol (ref=Never)**			
Past	0.55	0.33-0.93	0.074
Current	0.95	0.63-1.44	0.824
**Comorbidity (ref=No)**			
Diabetes	1.63	1.12-2.39	0.023
High cholesterol	1.38	0.95-2.01	0.179
Hypertension	2.14	1.49-3.07	<0.001
Cardiovascular	1.36	0.86-2.15	0.308
Chronic respiratory	0.87	0.54-1.39	0.805
Chronic kidney	0.83	0.41-1.67	0.712
Chronic liver	0.66	0.25-1.72	0.723
Cancer	1.56	0.87-2.81	0.275
**Symptoms (ref=No)**			
Pain	0.41	0.29-0.59	<0.001
Jaundice	5.06	3.37-7.59	<0.001
Weight loss	2.25	1.59-3.19	<0.001
Nausea	0.58	0.4-0.84	0.008
Vomit	0.44	0.3-0.67	<0.001
Diarrhoea	0.61	0.4-0.92	0.038
Constipation	0.8	0.52-1.22	0.583
Fatigue	2.18	1-4.77	0.1
Loss of appetite	1.05	0.43-2.57	0.917
Pruritus	4.34	1.21-15.6	0.049
Steatorrhoea	2.21	0.56-8.64	0.493
Change in bowel habits	1.38	0.91-2.08	0.237

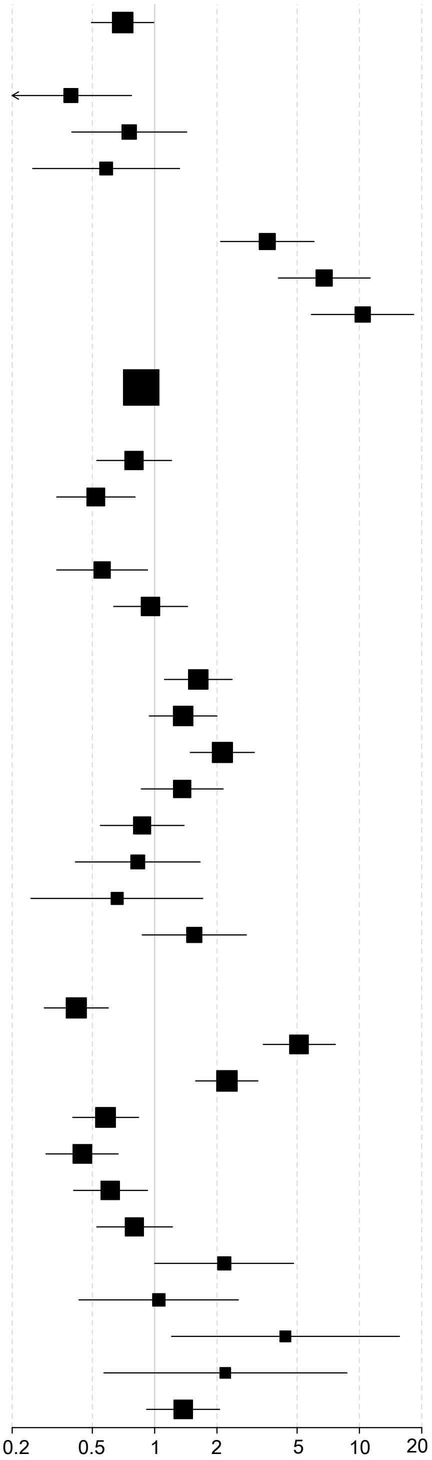

Supplementary Figure S1. Forest plot showing crude association between study variables and odds of PDAC in comparison to non-malignant pancreatic disease group. The odds ratio (OR) and 95% confidence interval (CI) are derived from logistic regression model. The reported p values are corrected for multiple testing via Hochberg method.

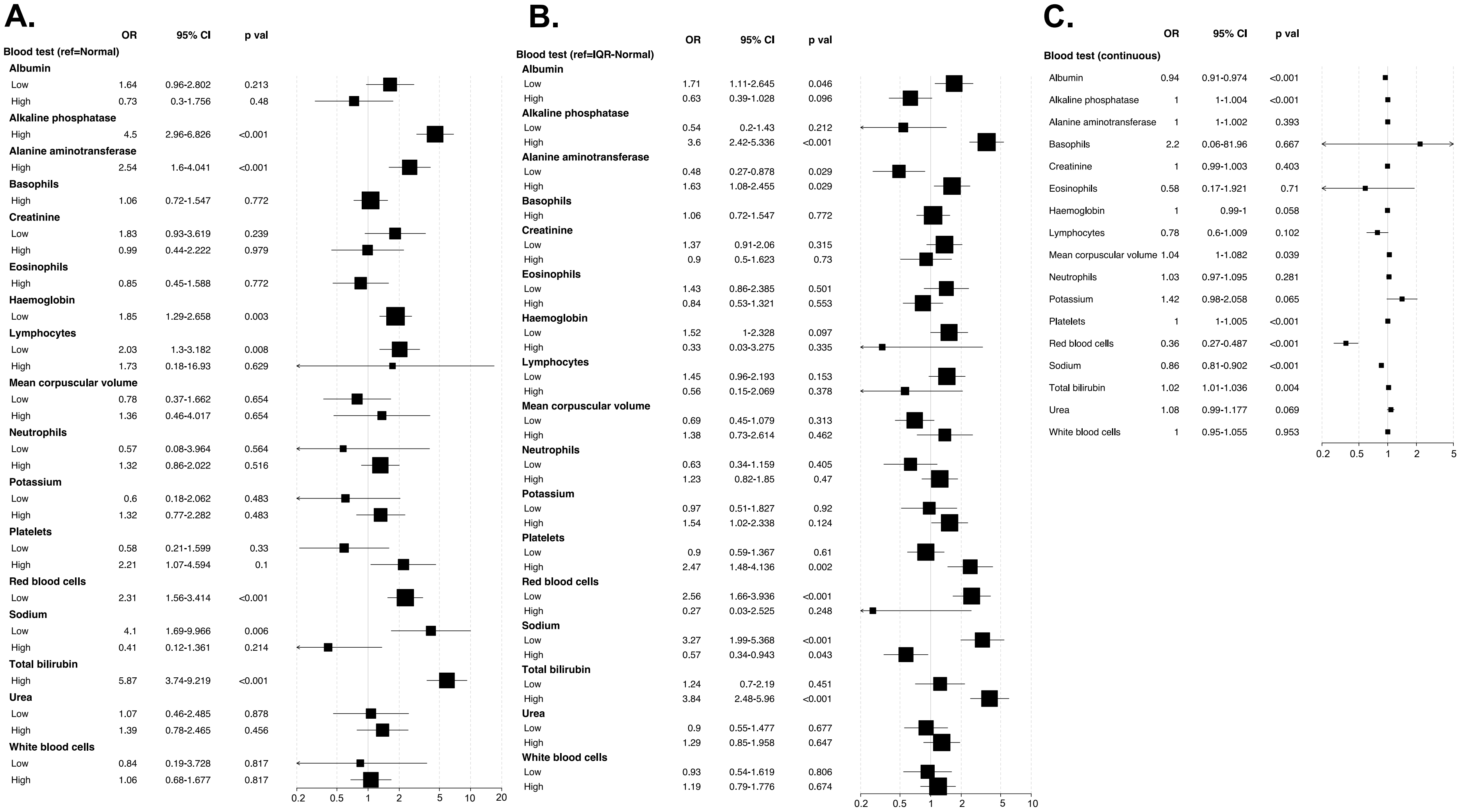

Supplementary Figure S2. Forest plot showing crude association between blood test result variables (a: categorical with known normal range as reference; b: categorical with interquartile of known normal range as reference; c: continuous) and odds of PDAC in comparison to non-malignant pancreatic disease group. The odds ratio (OR) and confidence interval (CI) are derived from logistic regression model. The reported p values are corrected for multiple testing via Benjamini-Hochberg method.
